# Equipoise in action: a qualitative investigation across six pragmatic randomised controlled trials (RCTs)

**DOI:** 10.1186/1745-6215-16-S2-P115

**Published:** 2015-11-16

**Authors:** Leila Rooshenas, Daisy Townsend, Julia Wade, Caroline Wilson, Jane Blazeby, Jenny Donovan

**Affiliations:** 1 University of Bristol, Bristol, UK

## Background

Research indicates that clinical recruiters often face conflict between their knowledge of clinical equipoise underpinning the RCT, and their instincts for/against eligible patients’ suitability for particular trial interventions. Little is known about whether or how equipoise is conveyed during recruitment practice. We investigated how equipoise was communicated by clinicians in recruitment consultations across six RCTs.

## Methods

123 consultations from RCTs across several specialties were audio-recorded and 29 clinicians were interviewed. Data were analysed using constant comparison techniques and content analysis. We focused on recurring practices that supported or undermined equipoise, and compared these to clinicians’ reported practices.

## Results

Though explicitly articulated in most - but not all - consultations, there were ‘within-trial’ variations in how clinicians expressed equipoise. This sometimes resulted in treatments being presented as superior/better-established from the outset. Some recruiters then maintained equipoise by presenting treatments as equally appropriate for the individual patient; others aligned individual patient characteristics with particular treatments, thus disrupting equipoise. Equipoise occasionally unravelled through clinicians’ subtle treatment recommendations, often in response to patients’ uncertainties or requests for recommendations. Though uncommon, some recruiters explored patient preferences, enabling them to restore equipoise if views were based on misconceptions. Interviews revealed recruiters were generally unaware of their practices that undermined equipoise.

## Conclusion

Equipoise is a fragile concept that can be supported or undermined by recruiters’ practices, though these may be unwitting. Clinicians should reflect on personal biases and be supported in developing strategies to maintain or restore equipoise, especially in response to patients’ uncertainties and/or misconceptions.

